# Formation and development of nanometer-sized cybotactic clusters in bent-core nematic liquid crystalline compounds

**DOI:** 10.3762/bjnano.9.121

**Published:** 2018-04-25

**Authors:** Yuri P Panarin, Sithara P Sreenilayam, Jagdish K Vij, Anne Lehmann, Carsten Tschierske

**Affiliations:** 1 Department of Electronic and Electrical Engineering, Trinity College, University of Dublin, Dublin 2, Ireland; 2 School of Electrical and Electronic Engineering, Dublin Institute of Technology, Dublin 8, Ireland,; 3 Institute of Chemistry, Martin Luther University Halle-Wittenberg, 06120 Halle, Germany

**Keywords:** bent-core liquid crystals, biaxiality, clusters, dielectric spectroscopy

## Abstract

Two homologue achiral bent-core liquid crystals (LCs), BCN66 and BCN84, in their nematic phases are studied by dielectric spectroscopy in the frequency range 10 Hz–10 MHz. In each of these compounds, two relaxation processes are identified and assigned to (i) collective dynamics of molecules in nanometer-sized cybotactic clusters and (ii) individual molecular relaxations, in the ascending order of frequency of the probe field. The temperature and the bias electric field dependence of the dielectric strength and relaxation frequency for these processes are shown to give rise to sharpness in cluster boundaries, increased size and volume fraction in the LC nematic phase. The effect of the bias field on the LC cell is similar to reducing its temperature; both variables increase the cluster size and volume fraction and give rise to sharp cluster boundaries. The findings confirm that dielectric spectroscopy is a powerful and an extremely useful technique to provide a deeper understanding of the mechanism of cybotactic cluster formation in the isotropic liquid and the nematic phase of LCs as a function of temperature and the bias field.

## Introduction

Biaxial liquid crystals (LCs) exhibit supramolecular structures with long-range orientational ordering of the long and the short molecular axes but without a long-range translational order. The optical switching by rotation around the long molecular axes is much faster and easier than switching by rotation around the short axes. In 1970, Freiser predicted that the long and flat LC molecules could form a biaxial nematic phase (Nb), and furthermore, a second-order transition could occur from the uniaxial nematic (Nu) to biaxial nematic (Nb) phase as the temperature of the LC is reduced [1]. Since this theoretical prediction by Freiser, the possibility of the biaxial nematic phase in LC systems was extensively explored theoretically in the condensed phase of the board-shaped molecules, that is, rectangular blocks of dimensions length (*L*), breadth (*B*) and width (*W*) [2], which could exhibit the biaxial nematic phase. The optimal dimensions for a biaxial phase to exist were found to be 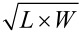 which is the molecular shape of an ordinary brick. It was predicted that molecules with a smaller *B*-parameter (*B*→*W*) have a tendency to form a uniaxial nematic phase and shapes with a higher *B*-parameter (*B*→*L*) would have a tendency to form a uniaxial discotic phase. In general the biaxial system can be characterized by four order parameters [2]. Similar results were obtained by a Monte-Carlo simulation for biaxial particles via an anisotropic pair potential [3]. Such a system exhibits three phases: biaxial crystalline at absolute zero temperature, uniaxial nematic and a rotationally disordered isotropic phase. In spite of the several theoretical studies having been successfully carried out, consistent attempts in designing and synthesizing thermotropic board-shaped LCs with biaxial Nb phase existing over a range of temperatures have largely been unsuccessful. Attempts to obtain a biaxial nematic phase by lowering the temperature of an ordinary uniaxial nematic phase resulted in either a glass, a smectic and/or a crystal being formed. In spite of the several setbacks of not unambiguously obtaining a biaxial nematic LC, the subject continues to be of great scientific and technological interest [4] as it has the potential to obtain faster switching devices that incorporate such a material. Nevertheless the biaxiality was observed in systems other than the board-like molecules, such as a lyotropic LC [5] and LC polymers [6–7]. At least two low molar mass, thermotropic systems were initially found to have a biaxial nematic phase; these were tetrapodes [8–9] and bent-core (BC) LC systems with nematic phase [10–14]. Spontaneous biaxiality in the BC systems was confirmed in smectic LCs: orthogonal smectic-A (SmA)-like [15–18] and the tilted smectic-C (SmC)-like [19–21] phases, while the existence of a biaxial nematic phase with large enough macroscopic biaxiality was highly debatable. A major issue arises as to whether the measurable biaxiality in the nematic phase is spontaneous or whether it is entirely induced by the electric field or surface effects [22]. As this issue is unresolved, the topic continues to be highly debated among the scientific LC community.

A number of bent-core systems in their nematic phase were studied during the last decade and some of these were shown to exhibit biaxiality on a macroscopic scale using X-ray diffraction (XRD) [12–13] and nuclear magnetic resonance (NMR) [14]. In most studies, however, the long axes of the molecules were aligned by surface treatment and one of the short axes was also aligned by the electric field. In more recent studies, the biaxiality of the sample studied was confirmed [3–5] by electro-optical switching [23], polarizing infrared spectroscopy (PIR) [24], polarizing optical microscopy (POM) [25], Raman scattering [26–27], XRD [28–29], photon correlation spectroscopy (PCS) [27,30–31] and NMR [32–33]. Recently Kim et al. [29] carried out X-ray experiments on a bent-core system in its nematic phase. They aligned the long molecular axes by applying a strong magnetic field parallel to the director. They showed that only the microscopic biaxiality exists in its nematic phase and no evidence for the macroscopic biaxiality was found. Nevertheless they did not align one of the short axes. Their X-ray results raise an important issue as to whether or not degeneracy in rotation around the long axes needs to be broken by aligning one of the short axes or if the long axes need to be subjected to such a strong magnetic field. Can such a large field induce the orientational order parameter greater than its thermodynamic value? Nevertheless it is now clear that many of the characteristics of the nematic phase of the bent-core LCs arise from so-called cybotactic clusters (with partial smectic order) [30–31] and hence detailed investigations of clusters and reasons for their presence in nematic phase are essential for answering the pending questions. For example, the spontaneous biaxiality may be small but it may easily be enhanced by aligning one of the short axes by surfaces or by the electric field [7,13]. Cybotactic clusters were considered to exist in the nematic phase by de Vries for the first time [34], who showed these arising from the pretransitional aspects of the smectic layers being present in the nematic phase. Hence these are found to exist only at temperatures close to the nematic–smectic transition temperature. We surmise that the mechanism for the formation of cybotactic clusters in the bent-core LCs may differ from that in calamitics. In the former, the seeds of clusters are formed by the molecular shape and the strong intermolecular interactions arising from the large molecular dipole moments parallel (μ_║_) and normal (μ_┴_) to the molecule’s long axis. In calamitics, however, molecules are rod-shaped, and the dipole moment lies either parallel (cyanobiphenyls) or normal (difluoroterphenyls) to the direction of the mesogen. The intermolecular interactions provide a basis for the mechanism in the formation of microscopic cybotactic clusters/domains in the nematic phase with C2v or higher symmetries [35–36]. The clusters are composed of nanometer-sized tilted (SmC-like) or orthogonal (SmA-like) segments. The structure of the cybotactic clusters in the bent-core nematics were studied by XRD [37–40] and two types of clusters were observed. These are NcybC and NcybA, depending on whether the molecules in the layers relative to the layer normal in clusters are bent or not (SmC or SmA). The electro-optical [41–42] and cryogenic transmission electron microscopy (cryo-TEM) studies [43–44] provided strong support for the existence of the presence of smectic-like clusters on the microscopic scale in the nematic phase. From these studies, it would appear that the bent-core nematic LCs consist of distinguishable clusters of lower symmetry, which in turn are made up of the correlated regions of molecules arising from their short-range orderings. In this paper we studied the formation of short-range molecular orderings in bent-core nematics by dielectric spectroscopy.

Dielectric spectroscopy is a complementary technique to XRD used to characterize different phases and structures of LC phases such as twist-grain boundary SmA (TGBA) [45], antiferroelectric liquid crystals (AFLCs) [46], de Vries [47–49], bent-core molecular systems [50–51], etc. Here we use it to distinguish the polar phases from the nonpolar ones. The latter are not active in the dielectric spectra.

## Experimental

**Figure 1:**
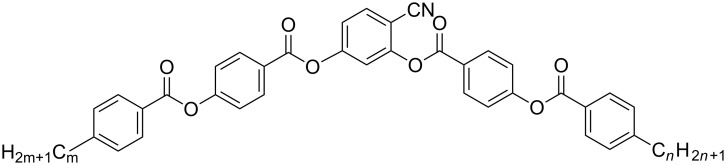
The molecular structure and phase transitions of two bent-core liquid crystal samples: BCN66 (*m* = *n* = 6): Cr 55 °C [37.1] N_cybC_ 101 °C [0.5] Iso [52] and BCN84 (*m* = 8, *n* = 4): Cr 62 °C N_cybC_ 96 °C Iso [51]. The transition temperatures were obtained on cooling under quasi-equilibrium conditions at a rate of ≈1 K min^−1^, Δ*H* was taken from DSC upon cooling (10 K min^−1^).

Dielectric measurements of the two bent-core LC compounds with controlled temperature and electric field in the frequency range 10 Hz to 10 MHz were performed. These compounds are members of a homologous series of 4-cyanoresorcinol. The molecular structure and the phase sequence of the compounds are shown in Figure 1. The first compound (BCN66) with a large flexoelectric polarization [52] is a member of the homologue series consisting of a bent core of five aromatic rings terminated by the two identical alkyl chains with variable chain lengths: C*_n_*H_2_*_n_*_+1_ where *n* varies from 2 to 14. The longer chain components form SmC-like B2 phases [53]. This series has been studied and characterized by polarizing optical microscopy and XRD [40] and displays a nematic phase with cybotactic clusters (N_cybC_) of the SmC type as explained above. The second compound, BCN84, has two asymmetrical alkyl chains of different lengths and is of the same molar mass as BCN66.

The dielectric permittivity measurements were carried out using a broadband alpha high-resolution dielectric analyzer (Novocontrol GmbH, Germany). The sample cells for dielectric measurements are constructed from glass substrates coated with indium tin oxide (ITO) with low sheet resistance (20 Ω/ ). This shifts the parasitic dielectric peak to a higher frequency due to the capacitance of the cell in series with the finite resistance of the ITO. The capacitance of the empty cell was measured. These measurements on the aligned liquid crystalline sample were carried out under cooling from 110 to 60 °C. The temperature was varied in steps of 0.5 °C under the application of a weak voltage of 0.1 V. The temperature of the cell with the samples was stabilized to within a range of ±0.02 °C.

## Results and Discussion

The bent-core nematic liquid crystals were previously investigated by dielectric spectroscopy for determining the elastic and viscosity constants [54], as well as the molecular dipole moment [55]. The short-range polar correlations among the neighboring molecules were revealed and the cybotactic clusters were established [35–36,48]. This was the first successful attempt for determining the cluster size by using the Maier–Meier equations using dielectric parameters such as anisotropic permittivity and the anisotropic Kirkwood correlation factor [56]. In that study [48] they used the reasoning that the relaxation time and the dielectric strength of the short-range polar correlations are proportional to the correlation length or the cluster size. The molecular mode corresponded to the dynamics of individual molecules. Therefore, the cluster size could be estimated from the ratio of the dielectric strength of the cluster and that of the molecular mode. The obtained cluster size agreed with the XRD results [38]. Here we apply the dielectric spectroscopic method to study the formation of the cluster structure and its size with variation of the temperature and the DC bias field.

**Figure 2:**
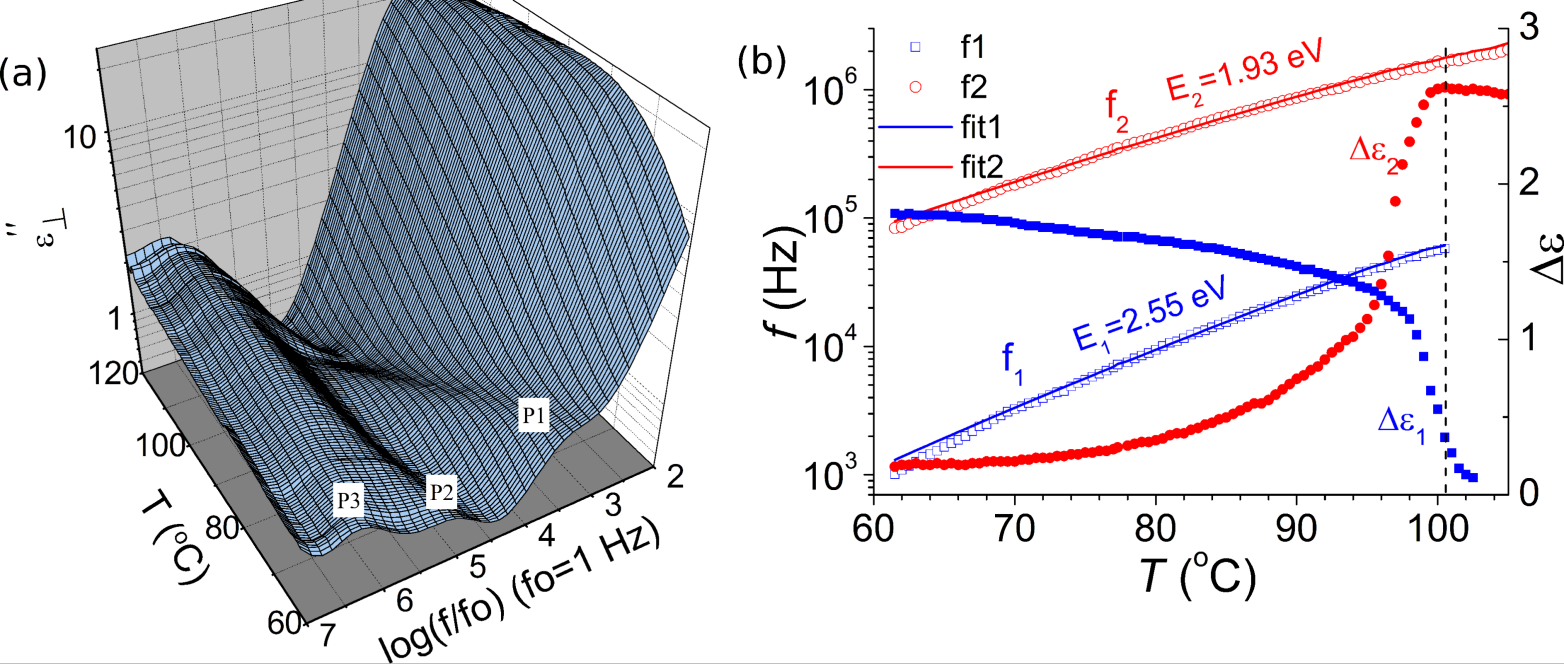
(a) Dielectric loss spectra (ε″) of a planar cell of cell thickness *d* = 7.8 µm in the frequency range 100 Hz–10 MHz and a temperature range of 60–120 °C. Measurements were carried out upon cooling the sample cell. (b) Temperature dependence of the dielectric strength Δε_1_ (filled blue squares) and Δε_2_ (filled red circles) and relaxation frequency *f*_1_ (open blue squares), and *f*_2_ (open red circles) are obtained by fitting the dielectric spectra to the Havriliak–Negami equation. Solid lines are fits to the Arrhenius equation, *f*(*T*) = *f*_0_ exp(−*E*_A_ / *k*_B_*T*), where *E*_A_ is the activation energy.

The dielectric spectra of both bent-core nematic LCs were studied by cooling the aligned samples from their isotropic to nematic phases, whereby the results were rather similar with each other. In this study, we focus on the BCN66 compound which had previously been studied by XRD [40]. This allows us to compare our results with XRD. The latter technique is regarded as the most appropriate technique in crystallography. Figure 2a shows the dielectric loss spectra of BCN66 as a function of frequency and temperature in a planar cell of cell thickness *d* = 7.8 µm. Three relaxation peaks are observed in the nematic phase during cooling from the isotropic state. The highest frequency peak (P3) is found to be temperature independent and is assignable to the finite resistance of the ITO-coated electrodes in series with their cell capacitance. The other two peaks are strongly temperature dependent and consequently assigned to the relaxation processes associated with the sample in the cell. The existence of two dielectric relaxation modes in the bent-core nematic phase was previously observed in [54–55]. The higher frequency is assigned to the individual molecular rotations around their short axes and the lower frequency is assigned to the fluctuation of cybotactic clusters themselves. This assignment is based on the evidence of cybotactic clusters being present in these materials [40]. The relaxation frequency is ≈100 times lower than that of the individual molecular modes [30–31].

### Temperature dependence of the dielectric properties

The dielectric spectra of BCN66 were analyzed by using the WINFIT software of Novocontrol GmbH. The dielectric data on the complex permittivity were fitted to the Havriliak–Negami (H–N) equation (Equation 1).

[1]
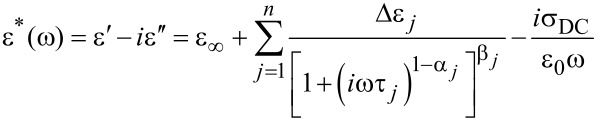


where ε***_∞_*** is the high frequency dielectric permittivity that depends on the electronic and atomic polarizability of the material, ω = 2π*f* is the angular frequency of the probe field, ε_0_ is the permittivity of free space, σ_DC_ is DC conductivity, τ*_j_* is the relaxation time of the *j*th process, Δε*_j_* is the dielectric relaxation strength, (1−α*_j_*) and β*_j_* are the symmetric and asymmetric broadening parameters that determine the distribution of the relaxation times of the *j*th process. In this case, the data are fitted to three processes, i.e. *n* = 3. The parameters values α*_j_* = 0 and β*_j_* = 1 correspond to the *j*th process being of the Debye type.

Figure 2b shows the temperature dependence of Δε*_j_* and *f_j_* obtained from fitting the dielectric data to Equation 1 over the entire temperature range of the nematic phase and temperatures in the isotropic phase close to the isotropic–nematic transition temperature.

Fitting of the dielectric spectra to the H–N equation shows that both of these processes are almost a perfect Cole–Cole type (i.e., β_1_ = β_2_ = 1). These are closer to the Debye type if α*_j_* approaches zero. The stretching parameter α*_j_* for these cases is found to lie in the range 0.05–0.25 depending on temperature. The higher frequency relaxation process (P_2_) exists in both nematic and isotropic phases and the strength first increases as the isotropic–nematic transition temperature is approached; this is followed by a significant reduction with a decrease in temperature in the nematic phase. This process is assigned to rotation of the individual molecules around their long molecular axes; the rotations are significantly hampered by a decrease in temperature in the nematic phase. The lower frequency relaxation process (P_1_) exists in both nematic and isotropic phases, where it is dielectrically detectable only ≈2 to 3 °C above the isotropic–nematic transition temperature. The latter observation arises as seeds of cybotactic clusters are formed in the isotropic phase. Small clusters have also been observed by dynamic light scattering (DLS) [57]. The strength of this process is observed to increase with a reduction in temperature in the nematic phase. This process is assigned to the collective fluctuations of molecules in the cluster. An increase in the size of the cluster leads to a higher dielectric strength as the temperature is reduced.

Based on these results, we find that the mechanism of cluster formation in bent-core systems is different from calamitics as first given by de Vries. In the latter case, these clusters would arise from the presence of pretransitional small fragments of smectic in the nematic phase described as a pretransitional effect. For this reason, these clusters in calamitics are observed closer to the nematic–smectic phase transition temperature by de Vries [34]. The XRD investigations of BCN66 in the nematic temperature range show two diffuse X-ray scatterings, wide-angle and small-angle. The small-angle scattering corresponds to the short-range periodicity of a group of molecules in clusters, whereas the large-angle scattering is characteristic of the mean distance between the individual molecules normal to their long molecular axes. The small-angle XRD signal splits into four peaks in the nematic phase of BCN66; this is thought to be due to the SmC-type clusters whereby their intensity increases as the temperature is reduced. Considering that polar and biaxial cybotactic clusters in the nematic phase exist, the lower frequency relaxation process is governed by the director fluctuations of molecules in clusters, whereas the higher frequency mode is associated with the dynamics of individual molecules outside the cluster.

These assignments agree with those obtained from the extended Maier–Saupe theory given by Droulias et al. [58], where they considered two different distribution functions, *f*′(θ) and *f*″(Θ). The distribution of the individual molecular directors relative to the cluster director, *n*^r^, is described by *f*′(θ), where θ is the polar angle between the long axis of the individual molecules and the cluster director. The distribution of the cluster director *n*^r^ relative to the macroscopic director *n* is described by *f*″(Θ), where Θ is the polar angle between *n*^r^ and the macroscopic director *n*. The order parameters *S*′ and *S*″ represent the degree of molecular orderings within clusters and the degree of ordering of clusters in the macroscopic sample, respectively. These are obtained from the following equations:

[2]
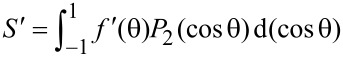


[3]
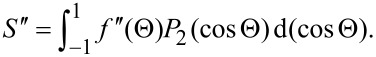


Here P_2_ is the second order Legendre polynomial in angles θ and Θ in Equation 2 and Equation 3, respectively. In the molecular field approximation (MFA), the orientational entropy of a cluster in the nematic phase is the sum of two contributions: the reorientation of the individual molecules and the fluctuation of the cluster directors with respect to the macroscopic director.

The temperature dependence of the relaxation frequencies *f*_1_ and *f*_2_ obey the Arrhenius mechanism. The activation energy corresponding to the processes P_1_ and P_2_ was found to be 2.55 eV and 1.93 eV, respectively. A higher value of the activation energy for the cluster compared to that of the individual molecules under cooling shows that the cluster slows down faster compared to the individual molecules. These frequencies are used to estimate the size of the cluster. When assuming that the relaxation frequency of the clusters depends on both the Arrhenius kinetic factor and its size, the average number of molecules present in the cluster (*N*_M_) can be estimated from the ratio of the two relaxation frequencies, *f*_2_ / *f*_1_. At 90 °C *f*_2_ / *f*_1_ = 864 kHz / 24.6 kHz ≈ 35. These results are verified by an XRD study [40], where the cluster size is estimated from the full width at half width maxima of the small-angle scattering. The cluster dimensions at 90 °C are found to be ≈2 times the length of a molecule in the longitudinal direction and ≈4 times the breadth of the molecules in the transverse direction, i.e., two layers each containing 16 molecules. The cluster size is therefore of approximately 32 molecules found from XRD, which in itself is in good agreement with the value (≈35) obtained from our dielectric spectroscopy results.

**Figure 3:**
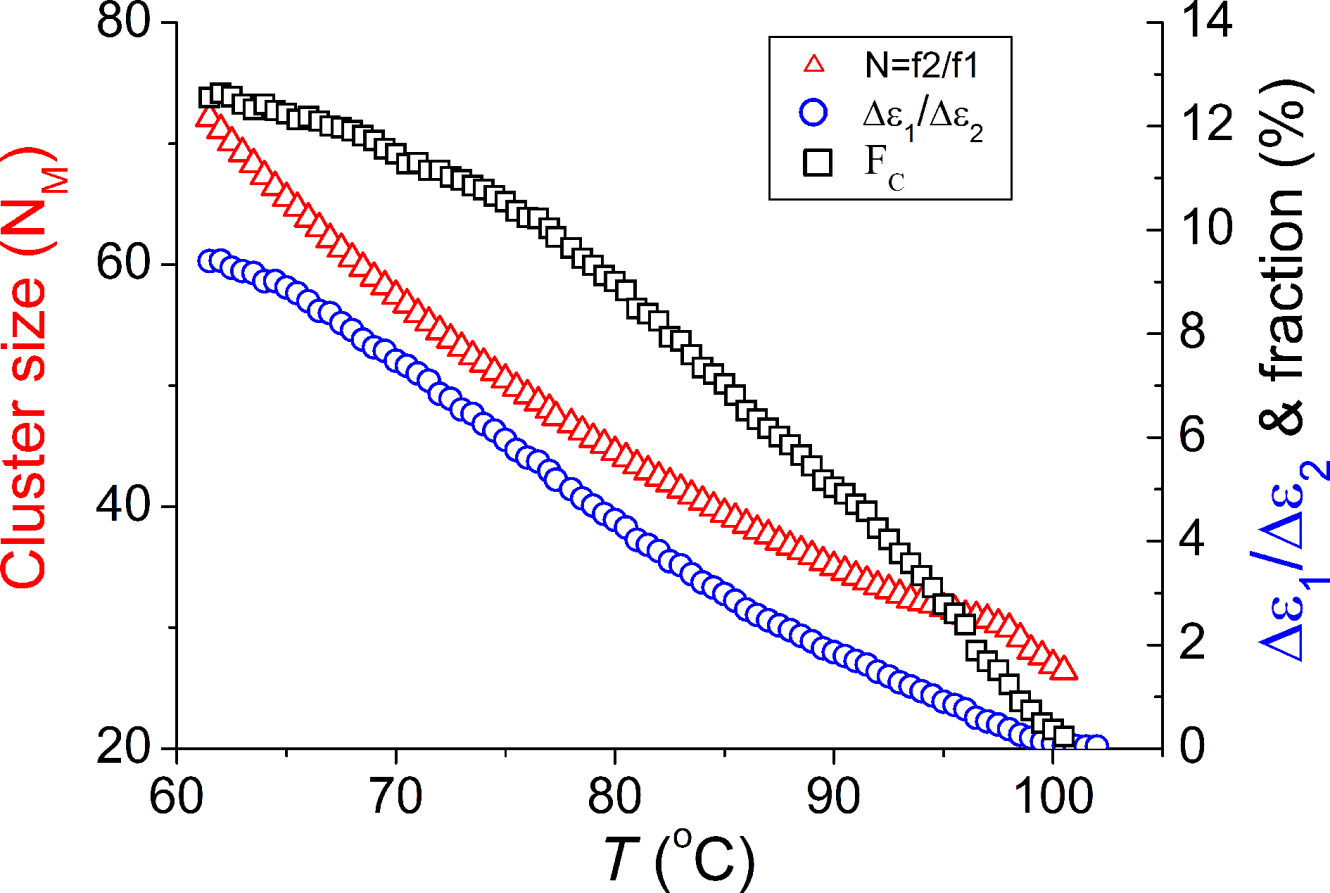
Temperature dependent cluster size (i.e., an average number of molecules involved in a cluster) (open red triangles), ratio of the dielectric strength, Δε_1_/Δε_2_ (open blue cirecles), the volume fraction of clusters (open black squares) in the bulk nematic phase, *F*_C_.

The number of molecules (*N*_M_) in a cluster increases upon cooling and reaches 72 (*f*_2_ / *f*_1_ = 83.8 kHz / 1.01 kHz) at 61 °C within the temperature range of the nematic phase (see Figure 3), again in agreement with XRD results [40]. This XRD study also shows that the intensity of the small-angle scattering strongly increases upon cooling and is interpreted as being due to an increase in the volume fraction of the clusters (i.e., the fraction of the molecules involved in clusters) [38]. The results from the dielectric studies are shown in Figure 3. The dielectric strength of the relaxation process P_1_ (Δε_1_) increases upon cooling from ≈0.11 (101 °C) to ≈1.85 at 65 °C (close to the transition to the smectic phase or to its crystallization), whereas the Δε_2_ of the process P_2_ decreases from ≈2.5 (100 °C) to ≈0.19 at 61 °C. The temperature dependence of the ratio of dielectric strengths Δε_1_/Δε_2_ is shown in Figure 3.

A higher Δε_1_ implies a larger number of molecules are present in the cluster. When considering that the dielectric strength of the cluster mode is proportional to its size and scaling it to the number of molecules in cluster (*N*_M_) we can estimate the volume fraction of the clusters occupied in the total volume as *F*_C_ = (Δε_1_/*N*_M_) / (Δε_1_/*N*_M_ + Δε_2_) = (Δε_1_/Δε_2_*N*_M_) / (Δε_1_/Δε_2_*N*_M_ + 1)) which is plotted as a function of temperature in Figure 3. The volume fraction of the clusters rises upon further cooling, which is reflected in the XRD study as an increase in intensity of the small-angle peak with decreasing temperature. It is also worth noting that the Cole–Cole stretching parameter of a cluster mode, α_1_, decreases upon cooling from 0.2 to 0.15, implying a narrowing of the cluster size distribution.

### Dielectric measurements with DC bias voltage

**Figure 4:**
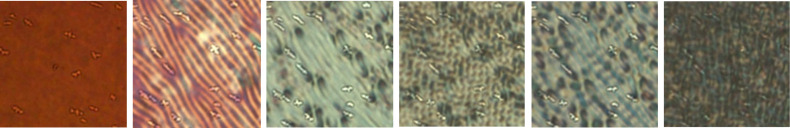
Polarized optical microscopy (POM) texture of a 7.8 µm BCN66 cell with an applied peak voltage of an AC square wave of 100 Hz. The original left-most texture is not affected by the AC voltage applied up to 10 V. Other textures were taken at the following voltages (from left to right): 11 V, 13 V, 20 V, 25 V, 30 V.

We investigated the dielectric spectra with applied bias voltage. Before carrying out the measurements, precautions were taken to avoid unwanted parasitic effects in the results. It is well known that application of the external electric field to nematic LCs might cause structural deformations of an initially homogeneous planar structure, i.e., it may cause a Freedericksz transition [59], create flexodomains, cause electro-convections, or a combination of these phenomena may result in a turbulent convection state. Depending on the frequency and the magnitude of the applied electric field, three types of electro-hydrodynamic instabilities have been observed in the BC nematics with negative dielectric and conductivity anisotropy [60–61]. During this exercise, we checked the effect of an external electric field on a homogeneously planar texture using polarized optical microscopy (POM). Figure 4 shows POM textures with different applied AC and DC voltages.

An application of a 100 Hz square wave voltage above an amplitude of 10 V causes turbulent convection with domains of different shapes appearing in the cell. A study of the electro-convection phenomena is beyond the scope of present work, however we find that a DC bias field up to 40 V does not affect the homogeneously planar structure created by alignment.

**Figure 5:**
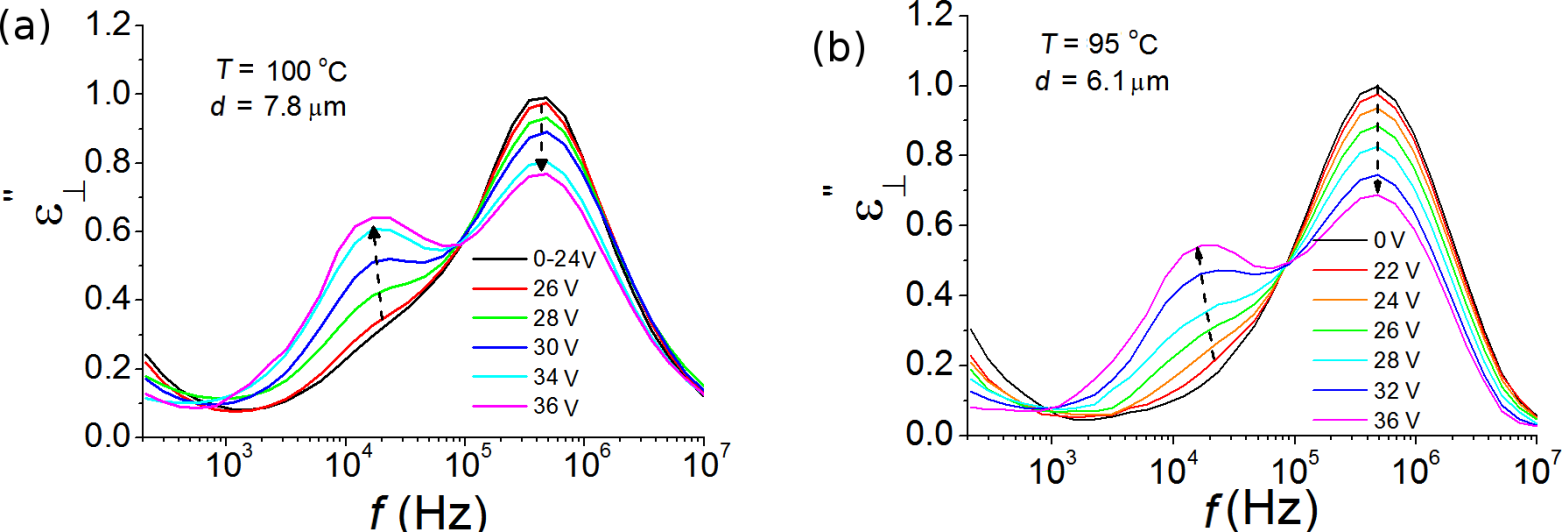
Dependence of the dielectric loss spectrum (ε″) on the DC bias voltage (0–36 V) in the nematic phase close to the N–Iso transition temperature for (a) 7.8 µm BCN66 cell at 100 °C and (b) 6.1 µm BCN84 cell at 95 °C [51].

**Figure 6:**
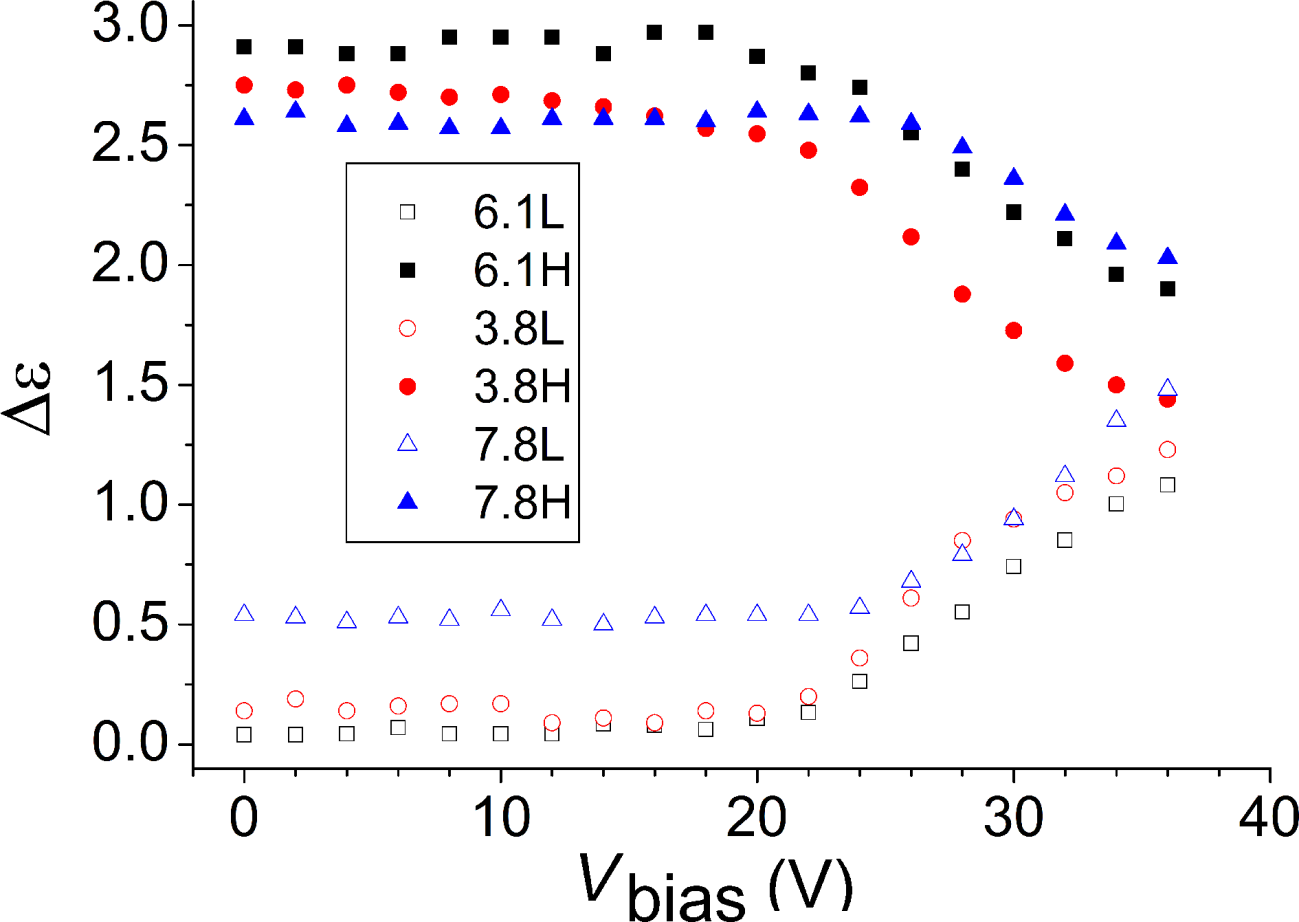
Dependence of the dielectric strengths (Δε) on the bias voltage for two processes (high frequency (H) and low frequency (L)) on the bias voltage in BCN66 (7.8 µm) and in BCN84 (3.8 µm and 6.1 µm) [51].

The dielectric loss spectra ε″ versus DC bias voltage were recorded for both BCN66 and BCN84 compounds at 1 °C below the isotropic–nematic transition, i.e. at 100 °C for BCN66 and 95 °C for BCN84 of two thicknesses, 3.8 and 6.1 µm. The dielectric loss spectra ε″ as a function of frequency for different bias voltages are plotted in Figure 5. The parasitic effect of ITO is subtracted for clarity. A comparison of Figure 5a and Figure 5b shows that the dependence of ε″ on the bias field are similar for both compounds. An application of the bias voltage up to 20–25 V does not affect the dielectric spectra. Above 25 V, however, the molecular relaxation process (P_2_) is gradually suppressed while the strength of the clustering process (P_1_) is significantly increased by increase in the bias voltage as shown in Figure 6. This means that the size of the cluster and consequently the volume fraction occupied by clusters in the bulk is reflected in an increase in the dielectric strength of the low-frequency (cluster) mode. On the other hand, an increase in the cluster size slows down the relaxation process, which is evidenced by the relaxation peak shifting to lower frequencies. To illustrate it clearly, we plot the dielectric spectra of the two individual relaxation modes deduced from the fitting procedure separately.

**Figure 7:**
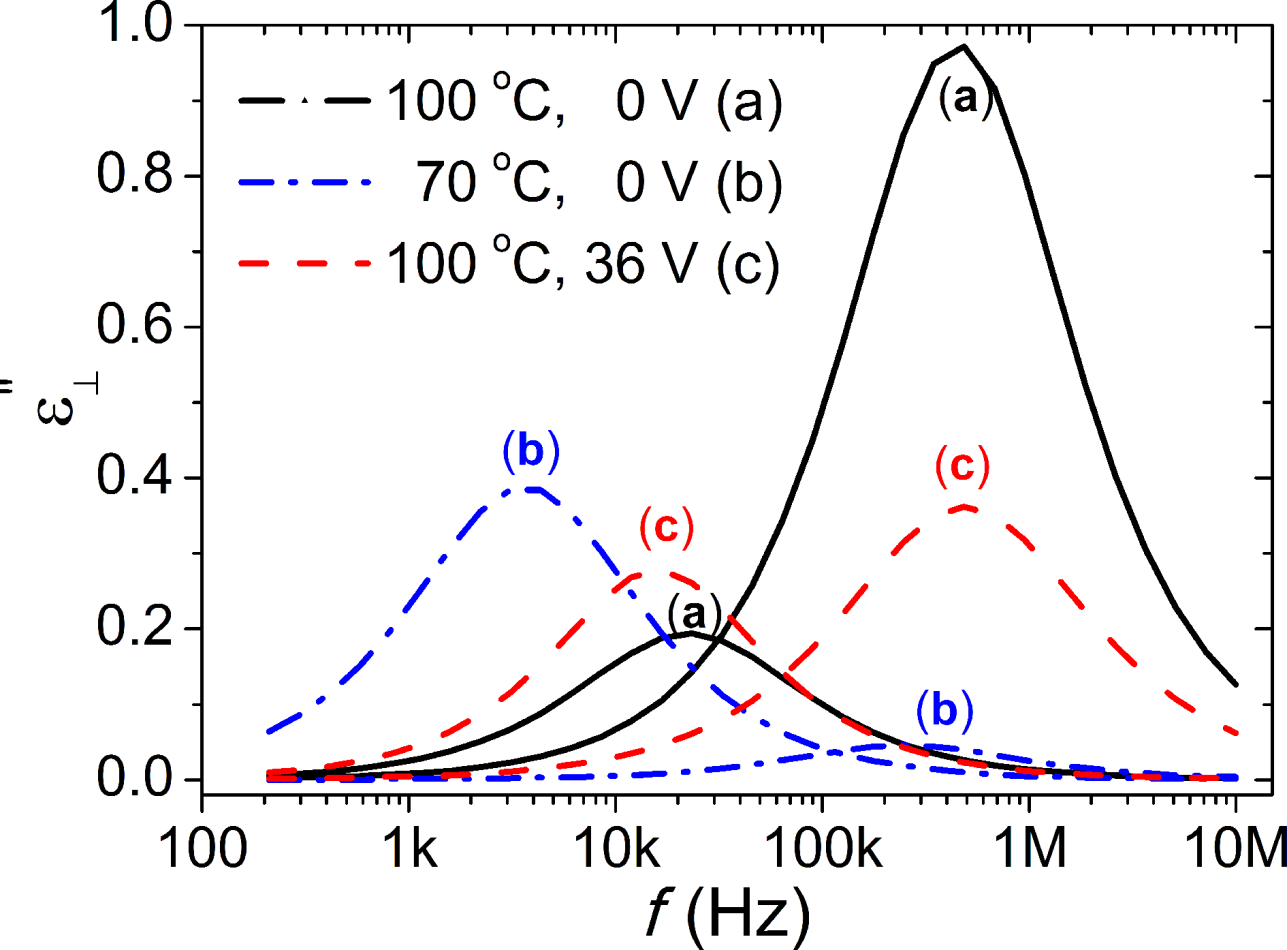
Effect of temperature and bias voltage on the two relaxation processes for BCN66: (a) solid black line 100 °C, 0 V; (b) dash–dot blue line 70 °C, 0 V; and (c) dashed red line 100 °C, 36 V.

Figure 7 shows the individual profiles of the two relaxation processes of BCN66 measured at two different temperatures and bias voltages. It should be noted that the relaxation frequency of the cluster mode decreases and its strength increases with decreasing temperature (compare Figure 7a and Figure 7b) and upon increasing the bias voltage (compare Figure 7a and Figure 7b). In other words, both reducing the temperature and increasing the bias voltage have similar effects on the cluster size. At a fixed temperature (100 °C in this case), the relaxation frequency of the individual molecular modes seen in Figure 7 is independent of the bias voltage but its strength is reduced significantly.

The effect of bias voltage on the dielectric strength and the relaxation frequency is similar to reducing the temperature – an increase in the bias voltage increases the cluster size and its volume fraction. This also narrows the distribution in the cluster size as shown by a decrease in α_1_ with decreasing temperature.

## Conclusion

We investigated two nematic bent-core liquid crystals with the same mesogenic core and molar mass but with different alkyl terminal groups terminated by: (i) symmetrical C_6_H_13_ and (ii) asymmetrical with different groups C_8_H_17_ and C_4_H_9_ using dielectric spectroscopy in the nematic and isotropic phases. Two relaxation processes are observed in the frequency range 10 Hz–10 MHz in the nematic phase. These are assigned to the individual molecular and the collective fluctuations of molecules in the cluster. The effect of temperature and the bias voltage on the dielectric spectra of both compounds were investigated. Fitting of the dielectric spectra to the standard Havriliak–Negami equation gives accurate values for the relaxation parameters, i.e., the dielectric strength and the relaxation frequency. A comparison of the temperature/voltage dependence on the dielectric parameters provides quantitative values of the size of cluster (the number of molecules in the cluster) and its volume fraction. The cluster size of these compounds deduced from dielectric spectroscopy is in good quantitative agreement with the results obtained from XRD [40] and also by dielectric spectroscopy in our previous paper [50]. This study elucidates the mechanism for the formation of the cluster structures and its transformations with temperature but also with the electric field. The clusters appear in the isotropic phase a few degrees above the isotropic–nematic transition temperature. The clusters start off initially from a small size in the isotropic phase by occupying only a small fraction of the total volume. The size grows with a reduction in temperature.

In addition, the transformation of clusters with decreasing temperature was studied previously by XRD. The dielectric technique gives additional information on the effect of the bias voltage on the cluster structure. The effect of the bias voltage on the cluster size and its volume fraction are found similar to the effects of reducing the temperature. A comparison of the dielectric behavior of both samples allows us to conclude that the cluster formation and transformation are almost identical for symmetrical and asymmetrical terminated bent-core systems; only the temperature range of the nematic phase for the two compounds is different. A deeper understanding of the cluster structure in the bent-core nematic phase in terms of its formations and transformations with bias voltage and temperature has been successfully obtained. This can form the basis by which the optical biaxiality in the nematic phase can be enhanced significantly by the electric field, enabling this finding to be used in practical devices.
